# Supplementary material for: Stress and anxiety among physicians during the COVID-19 outbreak in the Iraqi Kurdistan Region: An online survey
Source: PLoS One. 2021 Jun 29;16(6):e0253903. doi: 10.1371/journal.pone.0253903 (PMC8241071; doi:10.1371/journal.pone.0253903)
Supplement: Attachment — Submitted filename: Response to Reviewers.docx [file pone.0253903.s001.docx]

**PLOS One**

**Re: Submission of the revised manuscript**

Dear Editor,

Thank you very much for sharing the editors and reviewers’ comments and suggestions on the manuscript entitled “Stress and generalized anxiety disorder among physicians during the COVID-19 outbreak in the Iraqi Kurdistan Region: An online survey.” We thank them for these valuable comments and suggestions. We have found them very useful to improve the quality and clarity of the manuscript.

We have made the necessary revision by responding to the suggested comments. Please find below explanations to the revision made through point-to-point response to the comments. All the changes are in red color text.

Thank you very much for considering the revised manuscript.

Best regards,

**Comment**

The authors should provide the following information to the readers:

- What was the original language of the stress and GAD-anxiety instruments?
- What language or languages were used in the questionnaire?  Were the language or languages the primary languages of the physicians?
- What were the primary languages of the 370 physicians in the study?
- If a translation was conducted, how was it done?  Specific information on the original translation, back-translation into the original language, number and language backgrounds of those in creating the translation and/or any pilot/feasibility study of the original translation should be added.
- If translations were used, were the translated versions of the “stress” and “GAD anxiety” instruments re-standardized?  Is so, were they re-standardized using a clinical sample and/or non-clinical sample?

Author response:

Thank you very much for this comment. Details are now provided about using the original English language of the instruments (Page 8, 2^nd^ paragraph). There was no need to translate the instruments into the local Kurdish language as it was easy for the physicians to respond to the English instruments. As their entire medical education was in the English language, it is easier for them to use the English language in these instruments related to their health conditions.

**Comment**

Using a GAD-based instrument to assess anxiety may be problematic in certain ways, i.e.:

The authors have chosen only one of several manifestations of anxiety, i.e., Generalized Anxiety Disorder or GAD.  Why did they decide not to include other manifestations of anxiety, such as Posttraumatic Stress Disorder or PTSD (which occurs considerably more often than GAD in many stressed and most traumatized populations), specific phobias (which can be as common as GAD in stressful or traumatic settings), social phobia, panic disorder, and obsessive-compulsive disorder or OCD?  The absence of other anxiety-related experiences would comprise a limitation to a study having generic “anxiety” in the title (rather than “GAD-anxiety”).

Authors response:

Thank you for this important comment. This aspect of using only one manifestation of anxiety has been highlighted at the end of the discussion section and is considered a limitation to this study (Page 20, 2^nd^ paragraph).

**Comment**

Generalized Anxiety Disorder or GAD, when studied longitudinally over time, may morph into other disorders, some of them anxiety disorders (e.g., PTSD, Panic) and some other behavioral and psychiatric disorders (e.g., Substance Use Disorder, Pathological Gambling, Major Depressive Disorder).  This changing clinical phenomenon would be an advantage in a longitudinal study, in which the changes can be described.  In a cross-sectional study (such as this one), it is a limitation because we do not know how the GAD-anxiety experiences might have disappeared, advanced to GAD, or switched to another disorder.  Some anxiety disorders, such as OCD or phobias, may not begin with GAD but rather appear without a GAD entrée.

Authors response:

This limitation of a cross sectional study has been highlighted (Page 20, 3^rd^ paragraph), and the advantages of a longitudinal study in this respect have been emphasized.

**Comment**

Since there is no disability/dysfunction marker(s) in this study, we do not know whether the physicians were experiencing normal-range emotions, or symptoms of a kind that accompany disability/dysfunction.

- This absence of a distinction between normality and disability is a limitation to the study.
- In the absence of information on disability/dysfunction, these emotional experiences of stress or anxiety may be healthy under the circumstances  of a Pandemic.  Dysphoric experiences often compose a key motivating factor to change and adapt.
- Stated differently, a modicum of anxiety may be useful and realistic, given the circumstances.  Attempting to rid physicians of non-disabling anxiety may be counter-productive and undermine physicians’ efforts at adjusting to stressors and thereby reducing anxiety.

Authors response:

This issue of lack of disability/dysfunction markers has been highlighted as a limitation of this study (End of page 20 and beginning of page 21), and its details are discussed thoroughly.

**Comment**

Some people seek out challenges that involve anxiety experiences (such as those in GAD), but which they interpret as “exciting” (a positive valence) rather than “undesirable” (a negative valence).  Some people chose a medical profession for its challenge and excitement, as do people in a variety of other challenging occupations.  Without a longitudinal study, we do not know whether these cross-section GAD ratings are normative for physicians in this province or reflect external stressors (such as the Pandemic).  It would have also been helpful if the physicians might report their own subjective descriptors or concerns or aspirations as they enter the pandemic experience at this early phase.  The latter absence is also a limitation.

Authors response:

Thank you very much for raising this important and relevant aspect of anxiety. This limitation of not being able to distinguish a normative response or presence of stressor has been discussed in detail (Page 21, 2^nd^ paragraph).

**Comment**

During the period of the study, were there political or insurgent stressors that might have affected physicians in this province of Iraq?

Authors response:

Thank you. This is very much relevant to the Iraq Kurdistan Region situation. The influence of the political and economic situation during this period on the physicians has been discussed (Page 19, end of 2^nd^ paragraph).

**Comment**

The authors have made numerous statistical comparisons of associated variables at the .05 level.  For example, Tables 3, 4, 6, and 7 each have 6 comparisons.  Making numerous comparisons increases the rate of having a spurious comparison at .05 probability by chance alone.  The authors should consider using a correction factor to lower the level of probability.  For example, the Bonferroni correction (which equals .05/number of comparisons) would be .05/6 or .008 for the six comparisons in each of these tables.  Using this lower probability level would eliminate the significance of marital status in Table 6.

Authors response:

The probability level for the Chi square test has been changed to .008, as suggested. The interpretation of the results has been changed accordingly (Page 8, 4^th^ paragraph).

**Comment**

None of the data presented suggest to this reviewer that mental health interventions (e.g., counseling, anti-anxiety treatment) are warranted at this stage.  On the contrary, interventions should emphasize physicians’ ability to work safely and efficiently in the care of all their patients.  Their data indicate that generalists and those in special COVIC-19 units report the greatest GAD-anxiety.  Potential interventions that favor safety and efficiency might include:

- Generalists: triage for fever, symptoms, and/or exposure to COVID-19 by clinic staff member before seeing physician; patient and physician using mask and distancing in the office; using PPH equipment if exam or procedure is warranted (e.g., gloves, mask, possibly gown-cap-visor as needed).
- COVID-19 unit workers: need to follow PPH protocol to the letter; awareness that clinic co-workers have shown to be greater infection source in some settings as compared to patients as a source of infection.
- If the investigators decide eventually to utilize a counseling/mental health approach, I would be as concerned or more concerned about the potential alexithymic physicians who report minimal or no anxiety (and thus may not adhere to protocols and become COVID=19 carriers or active cases).

Authors response:

Thank you very much for this important suggestion. We completely agree with you. The issue of lack of enough justification for mental health interventions at this stage and the need for interventions that emphasize physicians' ability to work safely and efficiently has been discussed in detail (Page 18, last paragraph, and beginning of page 19).

**Comment**

The conclusions (stated in the Abstract) that “a need to establish mechanisms to reduce the risks of stress and anxiety and employ mental health coping interventions” are not supported by the data, which show demographic-professional associations with stress and GAD anxiety.  Since no interventions have been studied, these conclusions should be deleted.  The statement regarding “Additional research” is unnecessary and trite; it should also be deleted.

Authors response:

Thank you very much for this useful suggestion. The conclusion has been re-written to consider this comment (in the Abstract and main conclusion on page 21).

**Comment**

Abbreviations should be written out the first time they are used (e.g., COR, AOR).  Differences in computing the CORs and AORs should be specified.

Authors response:

The abbreviations are now written in full word the first time used (Abstract and page 9, 1^st^ paragraph)

**Comment**

New background information is presented for the first time in the Discussion on page 17, from line 281 to line 291 (beginning with “This could be” and ending with “a priority in the past”).  This important contextual information should be moved from the Discussion to the Background or Methods sections.  (New data should not appear for the first time in the Discussion.)

Authors response:

Many thanks for this useful note. This new background information has been moved to the Methods section (First paragraph of page 6), and a short necessary part of it was left in the Discussion section (page 18, 2^nd^ paragraph).

**Comment**

In several places the authors state that certain associations between demography-profession and stress and GAD-anxiety are “more prevalent” and then adding “not to a statistically significant degree.”  However, these associations did not meet the cut-off of .05 probability and therefore are not “more prevalent” by definition.  Thus, the following sentences should be deleted:

- Page 11, lines 192-194, “The prevalence was also higher….but not to a significant level.”
- Page 13, lines 210-214, “Severe anxiety was more prevalent…. but not to a significant level.”
- Page 14, lines 220-222, “The prevalence was also higher… but not to a significant level.”

Authors response:

All this text describing the non-significant association was omitted.

**Comment**

In the Discussion, on page 15, lines 232-233, the phrase “…the prevalence of stress was very high among the physicians…” is not clearly demonstrated by the data.  Considerably more data would be required to make this statement conclusively.

Authors response:

This sentence was revised to consider this comment (page 16, 1^st^ paragraph).

**Comment**

I assume that the stress scores and the GAD-anxiety scores are correlated with each other.  However, conducting a correlation between the two would nonetheless be of interest.  First, are both scores normally distributed (so that a decision can be made regarding use of Pearson versus Spearman correlation)?  Second, is the correlation a strong, close one, or is it weaker and more distant?  Are there many case examples of high stress with low anxiety and/or low stress with high anxiety?  If mental health services might be warranted, it would be in those showing a reverse correlation between stress and GAD-anxiety.  For example, physicians with alexithmia in need of treatment might have high stress/low anxiety.  Those whose anxiety disorder has been tripped off by minimal stress might stand out by virtue of low stress/high anxiety.

Authors response:

Thank you very much for this suggestion. We highlighted the limitation of not being able to conduct such an important correlation between stress scores and GAD scores (end of page 19 and beginning page 20) as two separate survey forms were used.

**Comment**

The accuracy of the sentence on page 17, lines 279-281 should be checked for accuracy; “In this study, the prevalence of stress was significantly higher among the physicians working in COVID-19 centers than other physicians, but the anxiety level did not differ significantly.  Table 4 shows no 95% C.I. difference in stress as a function of working in a COVID-19 center; and Table 7 reveals that those physicians working in a COVID-19 center reported more GAD-anxiety as measured by the adjusted odds ratio (AOR) of the 95% C.I.

Authors response:

Thanks for this note. This sentence was revised and corrected to reflect the actual finding (page 18, 2^nd^ paragraph).

**Comment**

Does the total population in the region amount to 370 at the time of the study?  This information is implied, but not clearly stated.  Was this the total number on a single day, or during 2.5 weeks of data collection?  For those trying to replicate this study in another setting, such details would be helpful.

Authors response:

Details about the physician population and sample are provided now (Page 6, 2^nd^ paragraph).

**Comment**

How were the authors able to elicit 100% participation in Part 1 and Part 2 data collection, but then lose 46% of participants in Part 3?  What might they have done to elicit greater participation in Part 3?  How important a limitation is the 46% loss of participants in Part 3? Were any additional data obtained?

Authors response:

Details about the response rate and the difference in response rate between the two surveys are provided (Page 9, first paragraph under Results and Page 19, last paragraph in Discussion).

**Comment**

It appears that the individual items in the stress and for anxiety instruments tap into severity in the individual items (10 for stress, 7 for anxiety), and the scale scores (0-4 and 0-3 respectively) tap into frequency.  If so, such information would be helpful to readers, who are likely to consider that all of these items occur at one time or another in all human beings; but what distinguishes individuals from one another is the severity and frequency of the stress or anxiety experience.

Authors response:

Thanks for helping with this useful clarification. Details of this aspect of the severity of symptoms and frequency are provided now (Page 7, last paragraph).

**Comment**

It is not clear how the authors determined what “stressor” or “GAD anxiety” scores would be assigned to various categories (such as “low-moderate-high” for stressors, and “not at all – several days – over half the days – nearly everyday” for GAD anxiety).  The distribution of the three stress levels in Table 3 seems to suggest that they employed a normal distribution since “moderate” percentages in the middle tended to contain about 2/3 of participants, whereas most “low” and “high” stressor groups contained about 1/6 of participants.  The GAD-anxiety distributions were similar, if “several days” and “over half of days” were merged into a single “middle” variable, which contained about 2/3 of most items.

Authors response:

Thanks for helping with the description of this procedure. Details of using the normal distribution to categorize the groups are provided now in the Methods section (Page 8, 1^st^ paragraph).

**Comment**

What are the rationales for grouping selected subgroups into single groups, as was done in Tables 4 and 7 for example?

Authors response:

The rationale for grouping each of the stress and anxiety into two groups only (dichotomous variables) is now provided (Page 8, 4^th^ paragraph).

**Comment**

On page 12, line 200, the use of the English “normal” presents some problems – in part because “normal” can means different things in English.  The latter include “average” (a statistical concept), healthy or non-pathological (a health/wellness concept), socially or culturally acceptable, ethically or morally correct, etc.  Since 9.5% is clearly not in the middle of a normal distribution, it is not “statistically normal.”  It may or may not be psychologically “normal.”  One might consider that those who report no anxiety may be “normal” in a wellness/health or culturally-desirable sense.  However, it is possible that reporting no symptoms may be undesirable or unhealthy.  For example, this group may alexithymic (i.e., unable to perceive their own distress, or minimizing their distress, or ignoring their distress), schizoid, lying, in denial, not recognizing the seriousness of the situation, etc. So, this small group may be abnormal in a variety of ways (i.e., statistical, health/wellness, most adaptive).

Authors response:

This description is now corrected (Page 13, 1^st^ paragraph) to avoid the word normal and the confusion it might cause.
