# Supplementary material for: Stress and anxiety among physicians during the COVID-19 outbreak in the Iraqi Kurdistan Region: An online survey
Source: PLoS One. 2021 Jun 29;16(6):e0253903. doi: 10.1371/journal.pone.0253903 (PMC8241071; doi:10.1371/journal.pone.0253903)
Supplement: Attachment — Submitted filename: Response to Reviewers.docx [file pone.0253903.s002.docx]

**PLOS One**

**Re: Submission of the revised manuscript**

Dear Editor,

Thank you very much for sharing the academic editor’s and reviewers’ comments and suggestions on the manuscript entitled “Stress and anxiety among physicians during the COVID-19 outbreak in the Iraqi Kurdistan Region: An online survey.” We thank them for these valuable and useful comments and suggestions. They helped us to improve the quality and clarity of the manuscript.

We have made the necessary revision by responding to the suggested comments. Please find below explanations to the revision made through point-to-point response to the comments. The changes related to the Academic Editor’s comments are in red color font, the changes related to the Reviewer 1 comment are in blue color, while the changes related to Reviewer 2 comments are in brown color font.

Thank you very much for considering the revised manuscript.

Best regards,

**Academic Editor Comments**

1. What was the sampling design and technique? Based on this were the obtained results valid? Were the findings generalizable to the target population? How was the sample size calculated?

Authors’ response:

Thank you very much for this important comment.

Details about the sampling design are provided (page 6 last paragraph and beginning of page 7).

Details about the sample size calculation are provide (page 6, last paragraph).

Details about the generalizability of the findings and the study limitation in this regard are provided (page 23, first full paragraph).

2. The study tools require elaboration in terms of their validity and reliability measures as observed in the current sample. This is required to understand the validity of the measurements.

Authors’ response:

Details about the validity and reliability measures of the study tools are provided (page 8, end of first paragraph and end of second paragraph).

3. Since this is an online survey, how did the authors control for response from outside the target population, because random social media share often presents with this difficulty, unless the questionnaire has control questions to restrict the survey among the target population only.

Authors’ response:

Thanks for this important note. Details about controlling for response from outside the target population are now provided (page 7, end of first paragraph).

4. What was the response rate? Was response rate accounted when calculating the outcome measures?

Authors’ response:

Details about the response rates are provided (page 10, first paragraph under Results section) and also it is emphasized in the limitations of the study (page 21, first full paragraph).

5. How was partial/ incomplete response handled in the study?

Authors’ response:

Details about avoiding partial or incomplete responses are provided (page 9 end of the first full paragraph).

6. Were the authors able to conclude that those responded and the non-responders were statistically similar in terms of their background characteristics? If yes, then this finding may be incorporated as a supplementary information. However, if not, then authors need to acknowledge this limitation as it again threatens the generalizability of the research.

Authors’ response:

This issue of inability to conclude that responders and non-responders have similar background characteristics is highlighted as a limitation of the study (page 23 end of the first full paragraph).

I recommend the authors to follow appropriate reporting guidelines for online survey research and modify accordingly.

Authors’ response:

The reporting guidelines are now followed as much as possible.

Optional recommendation:
The authors may consider dividing their methods section in some appropriate sub-headings. This will make the article even more comprehensive.

Authors’ response:

The methods section is now divided in to four sub-headings (pages 5-10).

**Reviewer #1:**

1. It is stated that there are 4 COVID 19 specialized centers. Whether all these centers were covered? Again, how many health centers and hospitals were covered in the study? These selected centers and hospitals constitute what proportion of the entire study settings in Iraqi Kurdistan Region? It has to be specified, since the title conveys physicians among Iraqi Kurdistan Region.

Authors’ response:

Thank you very much for this important comment. Details about the inclusion of the four COVID-19 centers and the number of hospitals and health centers are provided (page 7, first paragraph).

2. Regarding PSS: “The total scores of this measurement were interpreted as follows: 0-13 scores were considered low stress, 14-26 scores were considered moderate stress, and 27-40 scores were considered high perceived stress” – what is the reference?

Authors’ response:

The reference for the interpretation of the PSS score is provided (page 8, first paragraph).

3. Regarding GAD7: “0-4 scores were considered having no anxiety, 5-9 scores were considered mild anxiety, 10-14 scores were considered moderate anxiety, and 15-21 scores were considered severe anxiety” – what is the reference?

Authors’ response:

The reference for the interpretation of GAD7 score is provided (page 8, second paragraph).

4. “The survey questionnaire included three main parts” – it is mentioned that first part constitutes demographic variables and it is not specified about the second and third part? Are these PSS and GAD 7 respectively? Please specify.

Authors’ response:

The three parts are now mentioned together (page 7, first paragraph under Study tools sub-heading).

5. Please mention about the - Ethics committee approval statement, reference number and date of approval.

Authors’ response:

The number and date of ethical approved is provided (page 9, second paragraph).

6. “We employed a normal distribution to classify the participants into different categories of stress and anxiety based on their total scores in the two measurements. Stress was classified into three groups, while generalized anxiety disorder was classified into four groups” – on what basis three and four groups were created? Again, what is the reference?

Authors’ response:

Thanks for raising this concern. The part about normal distribution classification is removed and the distribution is now based on the tool guidelines and previous studies. References for such distribution are provided (Page 8, first and second paragraphs).

7. “AOR represents the value that has been adjusted for the other covariates, including confounders” – how did you get it from univariate analysis? To my knowledge it will come from multivariable regression analysis. Please clarify.

Authors’ response:

Thanks for this note. This was corrected (page 10, first paragraph).

8. “Normal and mild anxiety groups were combined, and moderate and severe anxiety were ............. with multiple logistics regression analysis” – delete the term multiple from here. Dependent variable was dichotomized only to have logistic regression analysis. Again the term would be ‘logistic’ instead of ‘logistics’.

Authors’ response:

Thanks for this correction. This was corrected and rephrased (page 9 and 10, first paragraph under statistical analysis).

9. Output of logistic regression is missing. What about model fitness, statistical significance of model, variation of dependent variables that can be explained from the independent variables etc.?

Authors’ response:

These details are provided in Table 4 (page 14) and Table 7 (page 17).

10. How independent variables were chosen in multivariable regression analysis?

Authors’ response:

Details about selecting independent variable in multivariate regression analysis is provided (page 10, first paragraph).

11. The term multiple in logistic regression is misnomer. It would have been multivariable logistic regression instead of multiple logistic regression.

Authors’ response:

Thanks for this note. This is corrected (page 10, first paragraph).

12. Table 1: Stress component: Age group & Job title – percentage is not coming to 100%. Please correct.
Authors’ response:

This occurred because of rounding. Now 2 decimals are provided for these (page 11, Table 1).

13. Table 3, 4, 6 & 7: Mention the chi-square statistic along with P value.

Authors’ response:

Chi square is mentioned now (pages 13-17, Tables 3, 4, 6, and 7).

14. Table 4, 6, 7: Mention years within parenthesis beside age group.

Authors’ response:

Years is mentioned now (pages 14-17, Tables 4, 6, and 7).

15. Table 4, 7: Footnote: Correct as ‘Crude odds ratio’ instead of ‘Crudes odds ratio’.

Authors’ response:

It is corrected (pages 14 and 17, Tables 4 and 7).

16. Table 5: Item 1 – percentage is not coming to 100%. Also check the other percentages for any corrections.

Authors’ response:

This is because of rounding. Two decimals are now provided to correct this (page 15, Table 5).

17. Table 2 & 5: Better to mention the number and percentages as No. (%) format to have smooth look instead of keeping in separate columns.

Authors’ response:

The format of No. (%) is changes as suggested (pages 12 and 15, Tables 2 and 5).

**Reviewer #2:** 

1. Applying PSS and GAD in separate google forms and analyzing them together is problematic and lacks validity.

Authors’ response:

Thanks for this important note. This limitation is now highlighted and discussed (page 21, second paragraph).

2. While using a scale, referencing a disorder as yes or no according to Normal distribution is not acceptable and flawed. Rather it should be validated first and then to be diagnosed as yes or no according the to the cutoff marks.

Authors’ response:

Thank so much for this important comment. The part about normal distribution classification is removed and the distribution is now based on the tool guidelines and previous studies. References for such distribution are provided (Page 8, first and second paragraphs).

3. It was written as 'The level of generalized anxiety disorder was measured based on the 7-item Generalized Anxiety Disorder scale.' GAD7 is a screening tool for GAD and can also be used for measuring Anxiety symptoms. So reframing this sentence(similar sentences are repeated many times in the manuscript) should be done.

Authors’ response:

This sentence and other similar sentences throughout the manuscript were reframed.
