# Supplementary material for: Stress and anxiety among physicians during the COVID-19 outbreak in the Iraqi Kurdistan Region: An online survey
Source: PLoS One. 2021 Jun 29;16(6):e0253903. doi: 10.1371/journal.pone.0253903 (PMC8241071; doi:10.1371/journal.pone.0253903)
Supplement: Attachment — Submitted filename: Response to Reviewers.docx [file pone.0253903.s003.docx]

**PLOS One**

**Re: Submission of the revised manuscript**

Dear Editor,

Thank you very much for sharing the academic editor's and reviewers' comments and suggestions on the manuscript entitled "Stress and anxiety among physicians during the COVID-19 outbreak in the Iraqi Kurdistan Region: An online survey." We would like to thank them for these valuable and helpful comments and suggestions, which helped in improving the manuscript's quality and clarity.

We have made the necessary revision by responding to the suggested comments. Please find below explanations to the revision made through a point-to-point response to the comments. The changes related to the Reviewer 1 comments are in red color font, while the changes related to Reviewer 2 comments are in the blue color font.

Thank you very much for considering the revised manuscript.

Best regards,

**Journal Requirements:**

Please review your reference list to ensure that it is complete and correct. If you have cited papers that have been retracted, please include the rationale for doing so in the manuscript text, or remove these references and replace them with relevant current references. Any changes to the reference list should be mentioned in the rebuttal letter that accompanies your revised manuscript. If you need to cite a retracted article, indicate the article’s retracted status in the References list and also include a citation and full reference for the retraction notice.

Authors’ response:

All the references were checked again. We could not detect any retracted paper cited in our manuscript. We only found that reference number 38 in the previous version of this manuscript (Goodwin 2015) had a problem as the journal (Dialogues Clin Neurosci) is no longer being published, and the website has now been closed permanently. However, and following Reviewer 2 comment no. 7, this reference was removed as part of removing unnecessary and not acceptable sentences.

As part of revising the manuscript based on the academic editor and reviewers’ comments, two references were removed by removing the unnecessary sentences (references 37 and 38 from the previous version of the manuscript). Two new references were added when discussing the low response rate (references 37 and 38 in the current version of the manuscript).

**Academic Editor Comments**

Authors must address all comments by reviewer 1. They must also provide clarification to comments 1,2,3,6, and 7 by reviewer 2. Authors should consider discussing about their study's overall response rate in their discussion section.

Authors’ response:

All the comments of Reviewer 1 were addressed as explained under each comment below. All the comments of Reviewer 2 were addressed, and necessary clarifications were provided under each comment below. The overall response rate is now thoroughly discussed in the discussion section (Last paragraph of page 21 and beginning of page 22).

**Reviewer #1:**

The term ‘Multivariate’ should better be replaced by ‘Multivariable’.

Authors’ response:

The term ‘Multivariate’ was replaced by ‘Multivariable’, as suggested (Page 10 under Statistical analysis and pages 14 and 17).

Table 1: Two digits after decimals are not desirable while showing distribution of the study subjects. Again the uniformity will not be maintained. Thus please keep single digit after decimal and modify accordingly.

Authors’ response:

All the two digit decimals were changed to a single digit, and uniformity of all numbers is now maintained (Table 5 on page 15, Table on pages 11 and 12).

**Reviewer #2:**

1. Clarify the sample size calculation and precision level. Why chosen 3.5% as precision, though conventionally we take 5% as precsion. Non response rate how much to be taken here?

Authors’ response:

We have used a more conservative and lower precision than the conventional 5% for the sample size calculation as the expected prevalence of stress was estimated to be within the upper 30% level (71.5%). It is generally recommended that when the rate or prevalence is close to the 25-30% of the exterminates at both sides (i.e., 25-30% and lower or 70-75% or higher), a lower precision or margin of error should be used for sample size calculation. If we have used a 5% precision, then the required sample size would have been 248 instead of 417, which is close to the sample responded to the anxiety component of our study. The reason for using a lower precision is now included in the manuscript (end of page 6 and beginning of page 7).

2. Translation of the questionnaire is important. Back translation and validation is required even for English speaking persons. Eventhough questions are having medical terminologies that just not justify the reasons of non medical persons not responding this question. Moreover PSS scale and GAD scale is used for Common people also. Restriction of response strictly for medical persons must be maintained

Authors’ response:

In the manuscript, we have now explained that the original questionnaire was in the English language, including the anxiety and stress measurement scales. As we also administered the questionnaire in the English language, there was no need to translate it or back translate it to the Kurdish language. We have also explained that all the obtained data sets were checked, and it is confirmed that all respondents were strictly physicians (Page 7, end of the first paragraph).

3. Exclusion criteria of study participants must be mentioned. Otherwise reasons of covid related stress and anxiety can never be established

Authors’ response:

We have now included the main exclusion criteria of the study (Page 7, end of the first paragraph).

4. Better to use the term univariate analysis because logistic regression is strictly for dichotomous outcome controlling confounding factors

Authors’ response:

The term ‘univariate analysis' is now used throughout the manuscript (page 10 under the Statistical analysis section).

5. Response rate 44% is unacceptable in generalised Anxiety disorder

Authors’ response:

The low overall response rate and its impact are now thoroughly discussed in the Discussion section (Last paragraph of page 21 and beginning of page 22).

6. In discussion part, writing justification of intervention should be modified. Counselling can be given..and covid protocol guidelines to be followed.

Authors’ response:

This paragraph was modified, and the need for intervention through counseling is emphasized (last paragraph of page 20, the conclusion section in the Abstract, and the main conclusion section on page 23).

7. There are many lines in the discussion which are not related with the topic of interest, hence commenting these lines is absolute unnecessary and not acceptable. For example:

a. If mental health services are warranted, it would be for the physicians showing a reverse correlation between stress and anxiety [37]. For example, physicians with alexithymia in need of treatment might have high stress with low anxiety. Those whose anxiety disorder has been tripped off by minimal stress may be characterized by low stress and high anxiety.

Authors’ response:

The discussion section was revisited, and the unnecessary lines and sentences were removed, including the above line and some other sentences (pages 21 and 22).

b. The findings of the current study do not justify mental health interventions such as counseling and the use of anxiolytic treatment or pharmacotherapy at this stage. - How authors came to this conclusion, please explain.

Authors’ response:

The above part of the discussion was revisited. We agree with the reviewer's comment that intervention is required, and we have included that in our first version of the submitted manuscript. However, the above sentence and non justification for mental health intervention were recommended by the previous academic editor or reviewer who initially handled this manuscript in the very first round of peer review. We have revisited the findings and the discussion section again and agree that interventions through counseling are needed at this stage based on the COVID-19 guidelines protocol. Thus, we have modified the above sentence and included the need for intervention in the manuscript (last paragraph of page 20, the conclusion section in the Abstract, and the main conclusion section on page 23).
